# The prevalence of positive rapid diagnostic test of hepatitis C virus infection in Ghana

**DOI:** 10.11604/pamj.2020.36.322.22490

**Published:** 2020-08-21

**Authors:** Masood Maleki Birjandi, Mahbobeh Oroei

**Affiliations:** 1 Polyclinic, Iranian Red Crescent Society, Accra, Ghana,; 2 Hearing Disorders Research Center, Loghman Hakim Hospital, Shahid Beheshti University of Medical Sciences, Tehran, Iran

**Keywords:** Hepatitis C virus, rapid diagnostic test, screening, Ghana

## Abstract

**Introduction:**

hepatitis C virus (HCV) infection is one of the most common viral hepatitis in Africa. Rapid diagnostic test (RDT) is a useful tool to identify antibody anti-HCV in point of care. In this study, we decided to determine prevalence of cases with positive rapid diagnostic test of HCV infection.

**Methods:**

this cross-sectional study was conducted in a polyclinic, Accra, Ghana. Using convenience sampling, 728 participants were screened with blood-based RDT and interviewed about personal risk behaviors for transmission of HCV. Data was entered in SPSS version 18 and analyzed.

**Results:**

there was 1.6% positive RDT in our participants. The mean age of them was 29.58 ± 12.31 years old that were younger than the participants with negative RDT (p: 0.027). The rate of positive test was 66.67% in women and 33.33% in men. There was a negative association between age and RDT positive (aOR: 0.91, 95%CI 0.85-0.96). The odds of positive RDT in married participants was 6.32 fold others after adjusting model (p: 0.014). There were no important risk behavior for HCV, except one person with history of contacting blood or needles.

**Conclusion:**

the risk of positive RDT has a reverse relationship with aging and also it has an increase in married individuals. Therefore preventive education and screening for HCV should be a priority in young and middle-aged adults because of more sexual activity.

## Introduction

Hepatitis C virus (HCV) is one of the most common cause of chronic liver infection that can lead to persistent HCV, cirrhosis (15-30%), hepatocellular carcinoma (2-4% annually), liver failure, and death [1, 2]. Almost a quarter of liver cirrhosis and hepatocellular carcinoma are attributable to HCV that are higher rate in endemic regions [3]. The prevalence of HCV is between 2% and 3% in global, and out of 70 million patients have active viraemia. The burden of HCV infection is significant in developing countries due to poor control management (screening, treatment and protective behaviors) [4, 5]. The first action fighting for HCV is early detection. The screening procedures of it are based on to discover antibody anti-HCV in the suspected individuals that including rapid diagnostic test (RDT) and laboratory-based immunoassay. The final purpose of screening is to confirm viremia and core antigen of HCV (HCVcAg) or HCV RNA in blood or body fluids after positive RDT [2]. World Health Organisation recommends RDT as point-of-care testing for large scale population. This test is a cost effectiveness, and feasibility test in screening of viral hepatitis [6-8] and can be blood or saliva-based that both have good sensitivity and specificity compared to gold standard test [9]. All patients with positive RDT should be investigated with nucleic acid test (NAT) to detect RNA of HCV, because of HCV infection clears spontaneously in 15-45% cases [10-12]. The main routes of HCV transmission include using needles or other equipment that are infected with the blood of a person infected with HCV, being born to a mother infected HCV, getting a tattoo or body piercing in an unregulated setting, infected blood products with HCV and sexual transmission [13].

The seroprevalence of HCV in Africa is more than other regions globally [3, 13]. It was estimated 3% (2.6%-3.5%) in Ghana, so that regional seroprevalence of HCV was 6.4% (4.2%-8.6%) in Accra [14]. One of the most predominant for HCV transmission, especially in western Africa is unsafe sexual contact with a person infected with HCV [15], but some studies showed sexual transmission in HCV infection is controversial [13, 16]. It is certainly that unsafe medical procedures are the main route of transmission HCV in regions with poor facilities [14, 17]. However, we decided to design this study to investigate seroprevalence of HCV among the patients referred to our polyclinic in Accra and to determine personal risk factors in them.

## Methods

This cross-sectional study was conducted on patients of polyclinic, Accra, Ghana in 2019. The sample size was estimated at least 385 using the prevalence estimation formula with 90% confidence level, 0.05% accuracy and 50% prevalence [18]. The inclusion criteria of the patients were as follows: both genders who were not the known case of hepatitis and were aged 18 years or more, outpatients of the polyclinic, and able in participating in this study. We prepared a questionnaire using comprehensive literature review for data collection. The content validity of questionnaire was conducted by two authors. The variables of questionnaire were about risk agents of HCV transmission, and sociodemographic information of the patients. Based on convenience sampling, 728 patients were investigated during one year. All were screened with blood-based RDT and then interviewed with questionnaire study. Data was entered in SPSS version 18 and the statistical tests such as Chi-square test, and independent t test, and also Mann Whitney test (for skewed data) were applied. Also we used univariate and multivariate logistic regression to find odds positive RDT. The significance level of statistical tests was set at below 0.05 and 95% confidence intervals were calculated for unadjusted and adjusted odds ratios. This study was conducted according to the Declaration of Helsinki and ensured the patients that their personal and medical information would be kept confidential. Informed consent was obtained from all patients. The study protocols were confirmed by the Health and Rehabilitation Deputy of Iranian Red Crescent Society in 2019 and were in line with the humanitarian services of the Iranian Red Crescent Society at polyclinic in Ghana.

## Results

We used a screening test for 728 patients from July 2019 through September 2019 at polyclinic, Accra. They consisted of male: 254(34.85%), female: 474(65.15%) and average age was 39.64 ± 16.72 years old that males were younger than females (37.94 ± 16.80, 40.55 ± 16.62 respectively, p: 0.017). The majority of the patients were non-smokers (97.94%), non-alcohol users (88.32%) and being literate (75.69%) (Table 1). Table 2 shows the percentage of identified risk agents of HCV in the patients. We had 145(19.92%) persons with a history of testing HIV, 74(10.16%) persons with testing HBV and 4(0.55%) with testing HCV in the past year. The most common risk agents in patients were respectively history of sexually transmitted infections (STI) (9.23%), blood transfusion (6.18%), body piercing/tattoo (5.63%), exposure to blood/ blood products/ needles (5.49%) and having multi-partner in the last year (3.30%). In screening of HCV with RDT, there was 12(1.6%) positive RDT. The mean age of them was 29.58 ± 12.31 years old that were younger than other patients (p: 0.027). The patients with positive RDT were 66.70% (n = 8) female, 66.70% (n = 8) married, 33.30% (n = 4) were illiterate, 66.70%(6 from 9) were self-employed and were no alcohol user and smoker and also had no medical history of diseases. There were no important risk agents of HCV in the patients with positive RDT, only one person had a history of contacting blood or needles. Respectively, two and one patients from them had a history of doing HIV and HBV tests that were negative responses. All patients with positive RDT were referred for counseling and treatment to more equipped centers. Table 3 shows relationships among some variables and gender. There was no significant relationship between gender and positive RDT (p: 1.00). Male patients had a higher rate of blood transfusion before 1992, multiple partners, a history of STD, and being imprisoned (p < 0.001). There was a significantly negative association between age and positive RDT in unadjusted and adjusted models (Table 4). In the adjusted model, odds of positive RDT in the married patients was almost 6-fold others (aOR 6.32; 95% confidence interval: 1.46-27.30).

**Table 1:** baseline characteristics of the patients (n=728)

Variable	Number (%)
**Education Level**	
Illiterate	177(24.31)
Primary	118(16.21)
Middle/High	346(47.53)
College/Above	87(11.95)
**Gender**	
Female	474(65.11)
Male	254(34.89)
**Marital status**	
Unmarried	314(43.13)
Married	344(47.25)
Divorced	28(3.85)
Widowed	42(5.77)
**Occupation**	
Unemployed	97(14.39)
Manual worker	100(14.84)
Employee	164(24.33)
Professional	55(8.16)
Self-employed	249(36.94)
Housewife	9(1.34)
**Tobacco consumption**	
No smoker	713(97.94)
Current smoker	13(1.79)
Past smoker	2(0.27)
Alcohol consumption	
No	643(88.32)
Yes	85(11.68)
**Medical history**	
Hypertension	48(6.56)
Diabetes	7(0.96)
Ischemic heart disease	3(0.41)
COPD	3(0.41)
Depression disorders	42(5.77)
Anxiety disorders	26(3.57)
HIV/AIDS	1(0.14)
HBV	3(0.41)

COPD: Chronic obstructive pulmonary disease

HBV: Hepatitis B virus, HIV: Human immunodeficiency virus

**Table 2:** the frequency percentage of risk agents for HCV infection in the 728 patients

Items	Number (%)
History doing of HIV test	145 (19.92)
History doing of HBV test	74 (10.16)
History doing of HCV test	4 (0.55)
Intravenous drug use	3 (0.41)
Intravenous drug use in the last 6 months	2 (0.27)
Using of illicit drugs(except injection)	7 (0.96)
Using of illicit drugs in the last 6 months	3 (0.41)
Having received blood or products before 1992	45 (6.18)
Haemodialysis patient	3 (0.41)
Body-piercing/ Tattoo	41 (5.63)
Being born from a HCV- infected mother	2 (0.27)
Exposure to blood or needles	40 (5.49)
Organ recipiet	3 (0.41)
Living with a HCV- infected person	7 (0.96)
Having multiple partners in the last 12 months	24 (3.30)
History of Sexually Transmitted Infections	67 (9.23)
Having a new partner during the past year	18 (2.47)
Using condom in a non-regular partner	51 (7.01)
History of hemosexual	3 (0.41)
Living with a STI-infected partner	14 (1.92)
History of surgical/dental procedure	16 (2.20)
HBV/HCV-infected individuals in first degree family	11 (1.51)
History of being imprisoned	9 (1.24)

HBV: Hepatitis B virus, HCV: Hepatitis C virus, HIV: Human immunodeficiency virus

**Table 3:** comparison of some variables based on gender

Variable	Male	Female	p-value
Positive RDT	4(33.3)	8(66.7)	1.000
HIV/AIDS	1(100.0)	0(0.0)	N.A
HBV	1(33.3)	2(66.7)	N.A
Illiterate	39(22.0)	138(78.0)	<0.001
Being married	124(36.0)	220(64.0)	0.584
Current smoker	8(61.5)	5(38.5)	N.A
Alcohol consumption	43(50.6)	42(49.4)	<0.001
Body-piercing/Tattoo	6(14.6)	35(85.4)	0.004
Having received blood or products before 1992	28(62.2)	17(37.8)	<0.001
Having multiple partners in the last 12 months	19(79.2)	5(20.8)	<0.001
History of Sexually Transmitted Infections	34(50.7)	33(49.3)	0.007
Having a new partner during the past year	13(72.2)	5(27.8)	0.002
History of being imprisoned	8(88.9)	1(11.1)	0.001
Intravenous drug use	1(33.3)	2(66.7)	N.A
Using of illicit drugs(except injection)	5(71.4)	2(28.6)	0.054
Intravenous drug use in the last 6 months	1(50.0)	1(50.0)	N.A
Using of illicit drugs in the last 6 months	2(66.7)	1(33.3)	N.A

RDT: Rapid diagnostic test , HBV: Hepatitis B virus, HIV: Human immunodeficiency virus NA: Not applicable The significant level less than 0.05

**Table 4:** univariate and multivariate logistic regression for predicting positive RDT

Variables	OR(95%CI)	P-value	aOR(95%CI)	P-value
Age	0.95(0.91-0.99)	0.041	0.91(0.85-0.96)	0.003
Gender				
Female vs. Male	1.07(0.32-3.59)	0.909	0.90(0.25-3.24)	0.884
Education				
Illiterate vs. other	1.56(0.46-5.27)	0.466	2.28(0.58-8.90)	0.235
Marital status				
Married vs. other	2.26(0.67-7.57)	0.186	6.32(1.46-27.30)	0.014
History doing of HIV test	0.80(0.17-3.69)	0.777	0.60(.11-3.32)	0.561
History doing of HBV test	0.80(0.11-6.29)	0.833	1.17(0.11-11.63)	0.891
Exposure to blood or needles	1.57(0.19-12.53)	0.666	1.58(0.18-13.70)	0.674

HBV: Hepatitis B virus, HIV: Human immunodeficiency virus, RDT:Rapid diagnostic test, OR:Odds ratio, CI:Confidence interval, aOR:adjusted odds ratio The significant level less than 0.05

## Discussion

In this study, we found 12 patients with positive RDT that the majority had no identified risk agents for HCV and were female and young. The odds of RDT positive in married patients was almost six-fold higher than other patients and with increasing age trend, its risk had decreased. Prevalence of positive RDT of our study was in line with Lokpo study [19] but it was different from other studies due to study population and age group. Apea-Kubi *et al*. [20] and Ephraim *et al*. [21] studied pregnant or non-pregnant women and reported higher positive test than us and Allain *et al*. [22] studied blood donors and found lower than.

Ghana as one of sub-Saharan Africa countries carries a significant portion of the prevalence of viral hepatitis, especially HCV infections, so that its prevalence was estimated higher than other regions of Africa except Burkina Faso, Benin, Cameroon, Gabon, and Angola [13]. HCV infection mostly transmite from blood and blood products (typically before 1990), unsafe injection and medical procedures, intravenous drug use, organ transplants, needle-stick injuries, body piercings/tattoo, and vertical transmission. The sexual transmission is controversial among heterosexual couples, but it is an important route in HIV positive men who have sex with men (MSM) and recreational drug users [16, 23]. HCV infection can be co-infection with HIV or HBV due to common risk behaviors. The prevalence co-infection HIV/HCV is lower than HIV/HBV. The co-infection of these viral diseases induce aggregative complications and side effects of antiviral therapies [24-26]. Co-infection of HCV with HIV was reported 5.7% in Platt´s study [5] and 0.4% in Loarec´s study [27]. In our study, there was no positive history of HBV or HIV in the patients with positive RDT. RDT is a feasible and rapid test for HCV infection that anti-HCV detects in blood or saliva samples of patients. This diagnostic tool has excellent sensitivity and specificity as compared to all HCV enzyme immunoassay antibody tests. Tang *et al*. reported sensitivity 97% and specificity 100% [9]. RDT has no cross-reaction with some immunoglobulins and antigens such as HBSAg, Anti-HBS, and rheumatoid factor [28], but may be false-positive in patients with schistosomiasis [29, 30]. Our study showed aging as a protective factor that it is explainable with decline sexual desire and activity [31], and also against married people have more risk. We found positive RDT in the patients with age before 60 years old and in female patients was more common than male patients similar to Lokpo’s study and Niu’s study [19, 32]. Based on our knowledge, it is improbable to transmit from blood products, because Ghana has a national policy for controlling blood-borne viral infections among blood donors [14]. One of the main transmit routes is unsafe medical procedures/injections that we didn’t have enough information for judgment.

We found history of STI as the most common risk agent in self-reporting of our patient, but Akyar *et al*. reported body piercing and infection at birth as common risk factors [15], that we didn´t find. In line with evidence, we had more prevalence of using illicit drugs as a risk factor in men than women [33, 34], but did not observe positive RDT in the illicit drug users and patients with history of STI. Despite the common transmission pathways among HIV, HBV, and HCV infections, our study population had the history of HIV test more than other viral tests. This finding shows that the most health programs of Ghana have been devoted to combating HIV/AIDS and paid less attention to HCV infection. National and international evidence from Ghana comprises an increasing concern to high prevalence of HCV infection and its outcomes and also risk of co-infection HIV and HCV [14, 26, 35], therefore it is suggested a targeted screening program for these viruses in a form integrated rapid diagnostic test. Our findings showed that men had more multiple partners and STI than women. It is necessary men should be had a faithful monogamous relationship and refrained from sexual risk behaviors, to reduce the direct transmission of HCV in women and also indirectly in vertical transmission. It is not currently available vaccination for HCV infection in the world, while antiviral treatment exists that can be considered based on the type of HCV genome. The best practice against HCV infection is doing two important actions that include: 1) early identification and treat infected patients, 2) promoting level of awareness and attitude about routes of transmission of HCV infection in community.

**Limitations:** there are three limitations to our study. 1) Our study was not conducted multicenter and we only investigated a part of the population of Ghana that was referred to our polyclinic; 2) we didn´t screen other viral diseases (HBV, HIV) together HCV; 3) female patients were not questioned or tested in terms of pregnancy. We suggest a study with multistage sampling in rural and urban communities in which indices of viral hepatitis are measured in various subgroups population with considering pregnant women and children.

**Funding:** this study was funded by polyclinic in Accra, Ghana (the Deputy of Health and Rehabilitation of the Red Crescent Society of the Islamic Republic of Iran).

## Conclusion

It seems that aging reduces the risk of positive RDT, in other words, married people with a middle or young age have a more likely positive test due to more sexual activity. Therefore comprehensive preventive education and screening with RDT are recommended in those adults as integration into the primary health care of Africa countries.

### What is known about this topic

- The prevalence of HCV is significant in developing countries as African countries;
- The rapid diagnostic test is a useful tool to identify HCV in point of care;
- The screening of HCV is cost-benefit in controlling its complications.

### What this study adds

- The probability of positive RDT decreases with aging;
- The probability of positive RDT is more in married individuals than others;
- The most common individual risk factor is a positive history of sexually transmitted infections.
